# Inflorescence dimorphism, heterodichogamy and thrips pollination in *Platycarya strobilacea* (Juglandaceae)

**DOI:** 10.1093/aob/mct278

**Published:** 2013-12-03

**Authors:** Tatsundo Fukuhara, Shin-ichiro Tokumaru

**Affiliations:** Faculty of Education, Fukuoka University of Education, 1-1 Akama-Bunkyo-machi, Munakata, Fukuoka, Japan

**Keywords:** Dichogamy, duodichogamy, heterodichogamy, Juglandaceae, *Platycarya*, thrips pollination

## Abstract

**Background and Aims:**

Unlike other taxa in Juglandaceae or in closely related families, which are anemophilous, *Platycarya strobilacea* has been suggested to be entomophilous. In Juglandaceae, *Juglans* and *Carya* show heterodichogamy, a reproductive strategy in which two morphs coexist in a population and undergo synchronous reciprocal sex changes. However, there has been no study focusing on heterodichogamy in the other six or seven genera, including *Platycarya*.

**Methods:**

Inflorescence architecture, sexual expression and pollination biology were examined in a *P. strobilacea* population in Japan. Flowering phenology was monitored daily for 24 trees in 2008 and 27 in 2009. Flower visitors and inhabitants were recorded or collected from different sexes and stages.

**Key results:**

The population of *P. strobilacea* showed heterodichogamous phenology with protogynous and duodichogamous–protandrous morphs. This dimorphism in dichogamy was associated with distinct inflorescence morphologies. Thrips pollination was suggested by the frequent presence of thrips with attached pollen grains, the scarcity of other insect visitors, the synchronicity of thrips number in male spikes with the maturation of female flowers, and morphological characters shared with previously reported thrips-pollinated plants. Male spikes went through two consecutive stages: bright yellow and strong-scented M1 stage, and brownish and little-scented M2 stage. The latter contained more thrips, synchronized better with the receptive stage of female flowers of the reciprocal morph and is probably the main period of pollen export.

**Conclusions Platycarya strobilacea:**

is heterodichogamous and thrips-pollinated, both of which are relatively rare conditions in angiosperms. In male spikes of *P. strobilacea*, there is probably a temporal decoupling of pollinator attraction and pollen export.

## INTRODUCTION

Dichogamy is a plant reproductive strategy in which flowers, inflorescences or individuals change functional sex, and has been interpreted as a mechanism to avoid inbreeding and/or male–female interference within an individual (Lloyd and Webb, 1986; Bertin, 1993). Among diverse dichogamous systems, heterodichogamy is a population-level dimorphism that involves two morphs that are synchronously and reciprocally dichogamous to one another: when the plants of one morph function as males, those of the other function as females, and vice versa (Lloyd and Webb, 1986; Renner, 2001). Heterodichogamy has been reported or suggested from 14 unrelated angiosperm families (Renner, 2001; Endress and Lorence, 2004; Wang *et al.*, 2012; see also Okamoto, 1987). A disproportionately large proportion of the documented species are economically useful plants that are often cultivated en masse, probably because heterodichogamy is more likely to be detected under cultivation because of the large numbers of individuals that can be easily assessed for temporal sex expression. Thus, additional heterodichogamous taxa should be discovered as more species are studied in detail.

Heterodichogamy is associated with various pollination modes, breeding systems, life histories and dispersal agents (Renner, 2001). In *Acer* (Sapindaceae: Gleiser and Verdú, 2005; Renner *et al.*, 2007) and *Alpinia* and allied genera (Zingiberaceae: Kress *et al.*, 2005), each of which includes multiple heterodichogamous species, the phylogenetic histories of heterodichogamy have been analysed. However, the evolutionary patterns were not very clear, partly because many species sampled for the phylogenies lacked information on the presence or absence of heterodichogamy. Furthermore, Renner *et al.* (2007) casted doubt on the distinction of heterodichogamy from labile sex expression in *Acer*, because some trees in a population changed sex expression across years (e.g. Asai, 2000) and because the morph ratio often deviated from 50 : 50 (e.g. Tal, 2009).

In some heterodichogamous species, the two morphs are reciprocal in the timing of pollen and stigma presentation during the day; most commonly, the synchronous and reciprocal sex changes at the individual level occur around noon every day during the flowering season. In the other species, the reciprocal sex change occurs once during a flowering season; the sex-changing morphs are either protandrous (PA; functionally male first) or protogynous (PG; functionally female first) at the individual level.

In the temperate tree family Juglandaceae, heterodichogamy, the presence of PA and PG morphs, was long ago documented in *Juglans* and *Carya* (Darwin, 1876: 390, 1877: 10; Pringle, 1879; Meehan, 1880) and recognized as a significant factor affecting nut production (Polito and Pinney, 1997). The inheritance of the PA and PG morphs has been reported for both genera in 1980s (Gleeson, 1982; Thompson and Romberg, 1985). In *Juglans*, relative timing of floral development (Polito and Pinney, 1997), phenology and morph ratio in wild populations (Kimura *et al.*, 2003; Bai *et al.*, 2006), and patterns of gene flow between and within morphs (Bai *et al.*, 2007) have been studied. In contrast, the mere presence or absence of heterodichogamy has not been examined in the less economically important Juglandaceae genera.

The east Asiatic genus *Platycarya* is particularly interesting because numerous characters are thought to be associated with the transition from anemophily to entomophily. Recent phylogenetic analyses have placed *Platycarya* in subfamily Juglandoideae, a well-supported clade comprising Caryinae (*Carya* and *Annamocarya*), Juglandinae (*Juglans*, *Pterocarya* and *Cyclocarya*) and *Platycarya* (Manos and Stone, 2001; Manos *et al.*, 2007). However, *Platycarya* possesses numerous floral characters that differ from the rest of the family (Manos and Stone, 2001, and references therein). In contrast to the other juglandaceous genera and related families (Rhoipteleaceae, Myricaceae, Betulaceae, Casuarinaceae and Ticodendraceae: Li *et al.*, 2004; Sauquet *et al.*, 2012) with typical wind-pollination syndromes, some of the unique characters of *Platycarya* suggest insect pollination (Endress, 1986). They include erect and showy male catkins emitting strong scents, stout and short stamen filaments, small and sticky pollen grains, relatively small stigmas, and flowering during the rainy, warm season. If *Platycarya* is insect pollinated, it would be an additional example of the transition from wind pollination to insect pollination, which is extremely rare in angiosperms (Friedman, 2011). However, no information exists on the pollination ecology of wild populations.

In the present study, we document heterodichogamy in a wild population of *Platycarya strobilacea*, and present evidence that suggests the species is pollinated by thrips.

## MATERIALS AND METHODS

### Study site

A population of *Platycarya strobilacea* Siebold & Zucc. was observed in Munakata, Fukuoka Prefecture, Kyushu, West Japan (around 33 °49′31″N, 130 °34′6·1″E). The trees grew between rice fields and low hills covered with deciduous forest dominated by *Quercus serrata* Murray. *Platycarya* trees flower in June, which is rainy and moderately warm in that area: monthly precipitation and average temperature at Munakata meteorological observatory (33 °48·5′N, 130 °32·3′E) were 235·0 mm and 21·2 °C in 2008, respectively, and 144·5 mm and 22·3 °C in 2009, respectively (data obtained from the Japan Meteorological Agency, http://www.jma.go.jp/jma/menu/report.html).

### Monitoring of flowering phenology

**Fig. 1.**
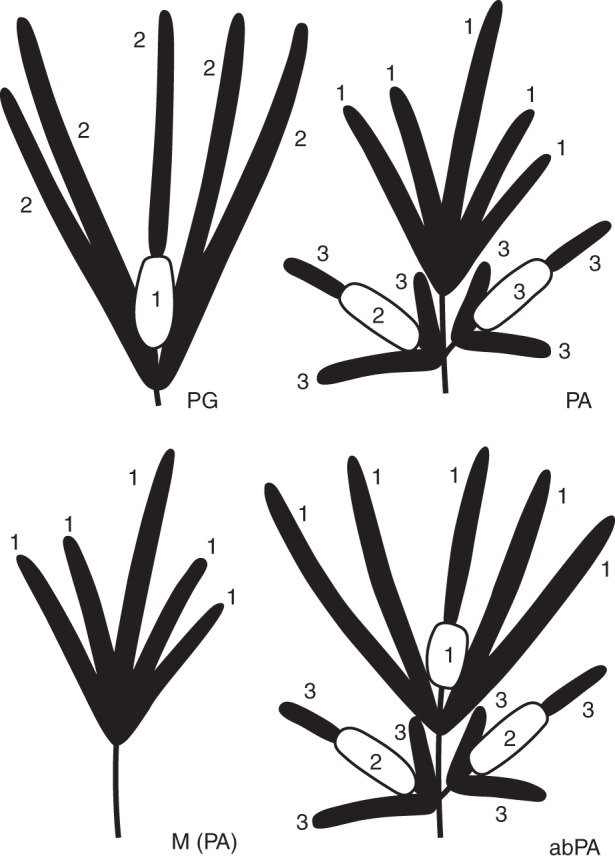
Inflorescence architecture of protogynous and protandrous morphs of *Platycarya strobilacea*. Male spikes or portions are shown as black bars and female portions as white oblong shapes. PG, protogynous inflorescence; PA, protandrous–duodichogamous inflorescence; M, protandrous functionally male inflorescence; abPA, ‘abnormal’ protandrous inflorescence. The numbers indicate the order of flowering.

**Fig. 2.**
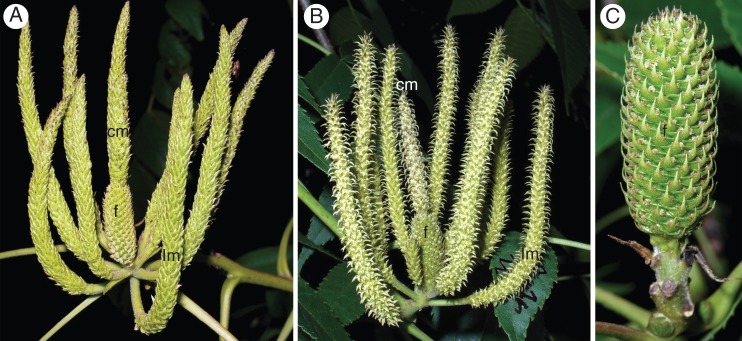
Flowering of protogynous (PG) inflorescences of *Platycarya strobilacea*. (A) An early-stage PG inflorescence. The female flowers are mature, while flowers of both central and lateral male spikes are enclosed in scale-like bracts. (B) A PG inflorescence at a later stage. Both female and male flowers are blooming; the male spikes have recurved bracts with whitish tips. (C) A PG infructescence. Only the female portion persists on the top of the main axis. f, female portion of a central androgynous spike; cm, male portion of a central androgynous spike; lm, lateral male spike.

**Fig. 3.**
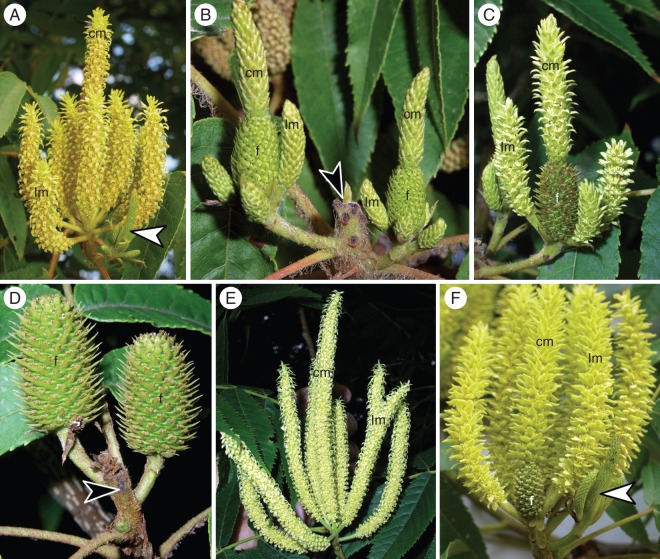
Flowering of protandrous (PA) inflorescences of *Platycarya strobilacea*. (A) An early-stage PA inflorescence, in which both central and lateral male spikes are mature. An undeveloped lateral cluster of spikes is visible to the bottom right (white arrowhead). (B) A PA inflorescence at a later stage, in which the female portions of the two lateral clusters are flowering. The male spikes of the main axis have fallen away and their scars are visible (black arrowhead). (C) A lateral cluster at the final stage, in which the female portion of an androgynous spike is developing fruits and the male spikes are flowering. (D) A PA infructescence, in which two female portions of androgynous spikes persist at the tips of lateral branches. The main axis has aborted (black arrowhead). (E) A functionally male PA inflorescence composed of the central and lateral male spikes with no lateral clusters. (F) An ‘abnormal’ PA inflorescence, which is similar to the typical one (A), but has a female portion basal to the central male spike. f, female portion; cm, central male spike or make portion of central androgynous spike; lm, lateral male spike.

In *P. strobilacea*, a cluster of male and androgynous spikes are borne at the apices of growing shoots (Figs 1–3). Male spikes are cylindrical. Androgynous spikes are composed of an apical cylindrical male portion and a basal female portion in which female flowers are spirally arranged on an oblong axis that resembles a pinaceous ovule cone. In this paper, a cluster of spikes terminal on a branch is termed an inflorescence. Both male and female flowers are small, lacking showy perianths, and each is subtended by lanceolate bracts (for a detailed description and interpretation of *Platycarya* flowers, see Li *et al.*, 2005).

In 2008 and 2009, five inflorescences per tree were marked prior to flowering. When fewer than five inflorescences were available, all were marked. Thirty-two trees flowered in 2008 and/or 2009; 98 inflorescences of 27 trees in 2008 and 95 inflorescences of 24 trees in 2009 were marked to observe inflorescence architecture and to monitor flowering phenology. In 2009 and 2010, the inflorescence architecture of an additional 15 trees nearby was examined.

Four male (M0–M3) and three female stages (F0–F2) were defined (Table 1); M1 and M2 were postulated to be when an inflorescence exports pollen and F1 when an inflorescence receives pollen. The stages of marked inflorescences were recorded daily around noon throughout the flowering season (30 May–2 July in 2008, 26 May–28 June in 2009, with the exceptions of 1, 11, 21 and 28 June in 2008).

**Table 1.** Flowering stages of *Platycarya* used to monitor phenology

Stage	Morphology
Male	
M0	Bracts partly recurved; no scent
M1	Bracts fully recurved showing white adaxial tips; strong scent; fresh yellowish anthers visible
M2	More than 50 % of anthers withered and brownish; no or weak scent
M3	Male inflorescence fallen
Female	
F0	Bracts partly recurved
F1	Bracts fully recurved; fresh whitish stigmas visible
F2	More than 50 % of stigmas brownish

### Phenology data analysis

In heterodichogamous populations, pollination may occur between the PA and PG morphs or within a morph, including via geitonogamous self-pollination. To estimate the degree to which dichogamous flowering increased the opportunity for between-morph pollination, the number of trees functioning as males and females on each date was calculated for each of the morphs as the sum of (the number of inflorescences at male maturity/the number of marked inflorescence in each tree) and the sum of (the number of inflorescences at female maturity/the number of marked inflorescence in each tree), respectively. Potential mating probability (PMP; Sato, 2002) for each combination of morphs was calculated by a modified equation of Sato (2002):

$$\eqalign{{\rm PMP}_{i,j} & = {\sum_d \left(R_{i,d} \times F_{i,d}\right) \over \sum_d \left(F_{{\rm PA},d} + F_{{\rm PG},d} \right)}\cr R_{i,d} &= {M_{i,d} \over M_{{\rm PA},d} + M_{{\rm PG},d}}\quad \hbox{when } M_{{\rm PA},d} + M_{{\rm PG}, d} \gt 0,\cr R_{i,d} &= 0 \quad \hbox{when } M_{{\rm PA},d} + M_{{\rm PG},d} = 0}$$

where *i* and *j* are morphs, i.e. PA or PG; *M*_*i,d*_ is the number of trees of morph *i* functioning as male on date *d*; *F*_*i,d*_ is the number of trees of morph *i* functioning as female on date *d*; and PMP_*i,j*_ represents the relative opportunity for the female-stage inflorescences of morph *j* to receive pollen from male-stage inflorescences of morph *i* throughout the flowering period.

To test the extent to which mating opportunity between morphs was enhanced by the temporal reciprocity in male and female maturity, PMPs were calculated based on a simulation in which the phenology of the PA morph was shifted to be 1–9 d earlier or 1–9 d later than the observed phenology. Simulated PMPs were then compared with observed values.

### Observations and counts of flower visitors/inhabitants

In 2009, the frequency, taxa and behaviour of insects visiting *Platycarya* inflorescences were recorded while we determined the stages of those inflorescences (approx. 50 h total observation time). On 1 and 10 June, the same observations were made at night (starting around 1900 h) for 5 h total.

In 2009 and 2010, spikes of PA and PG trees at various stages were collected to count thrips (see Table 4 for the number of spikes). Five spikes per inflorescence were collected and immediately put in a glass vessel filled with FAA (5 : 5 : 45 : 45 formalin/acetic acid/ethanol/water) or AGA (1 : 1 : 8 : 5 acetic acid/glycerin/ethanol/water) solutions for more than 12 h. Each vessel was shaken to dislodge thrips from the spikes into the solution, and the numbers of adults and larvae were counted. When there were more than 100 thrips, the solution volume was brought to 15 mL and 1·5 mL was pipetted for counting.

In 2010, 72 male spikes at stage M1 were taken from two PA and two PG trees. Each was immediately sealed in a plastic bag and stored at room temperature for 0, 2, 4, 6, 8 or 10 d, and then the number of thrips was counted for 12 spikes at each time as described above, after pouring AGA solutions into the bag. Because some spikes became dark coloured and partially collapsed after 6 d, the counts at 0, 2 and 4 d were used for comparison.

Because the distributions of thrips counts were extremely right-skewed and over-dispersed, a generalized linear model (GLM) assuming a negative binomial distribution and logarithm link functions was applied using R v. 2·14·1 (R Development Core Team, 2011). The effects of stage or storage period were analysed using an analysis of deviance (type II test) with *F* values.

## RESULTS

### Inflorescence architecture

In each inflorescence, flowers of the same sex matured synchronously, and each inflorescence functioned mostly as either male or female at any given time. The 47 observed trees were divided into PG and PA morphs based on their inflorescence architecture. The morphs also differed in the sequence of expression of male and female functions (Fig. 1; the order of flowering is numbered).

In the 22 PG trees, inflorescences were composed of a central androgynous spike (a basal female portion and an apical male portion) and up to ten lateral male spikes (Fig. 1, PG). In the PG inflorescences, the central–basal female portion flowered first (Fig. 2A), followed by both the lateral male spikes and the central–apical male portion (Fig. 2B). After flowering, the female portion formed an infructescence (Fig. 2C). Extremely rarely, a PG inflorescence was functionally male; the central–basal female portion was abortive, i.e. undeveloped and dark brownish.

In the 25 PA trees, inflorescences comprised a central male spike, up to ten lateral male spikes, and 1–4 lateral clusters of androgynous and male spikes (Fig. 1, PA). The central male spike tended to be longer than, and to protrude from, the lateral ones (Fig. 3A). The lateral clusters were similar to the PG inflorescence in having a central androgynous spike (a basal female portion and an apical male portion) and up to five lateral male spikes. The PA inflorescences were duodichogamous; that is, flowering in a male (labelled ‘1’ in Fig. 1, PA) – female (‘2’) – male (‘3’) sequence. The central male spike and its lateral male spikes flowered first, when the lateral clusters were small and in bud stages (Fig. 3A). After those spikes finished flowering and fell to the ground, the lateral clusters continued expanding. In the lateral clusters, the central–basal female portion flowered first (Fig. 3B), followed by both the lateral male spikes and the central–apical male portion (Fig. 3C). After flowering, 1–4 infructescences were borne laterally (Fig. 3D). Quite a few PA inflorescences lacked lateral clusters and were functionally male (Fig. 1, M; Fig. 3E). Within each tree, functionally male inflorescences were scattered among monoecious ones but were more frequent in lower and more shaded positions. The ratio of functionally male inflorescences to monoecious ones varied among trees and years. Sometimes, only functionally male inflorescences were borne (see below). Two of the 25 PA trees possessed ‘abnormal’ PA inflorescences, with a female portion at the base of the central male spike (Fig. 1, abPA; Fig. 3F) that flowered concurrently with the male spikes.

### Phenology and sex expression

**Fig. 4.**
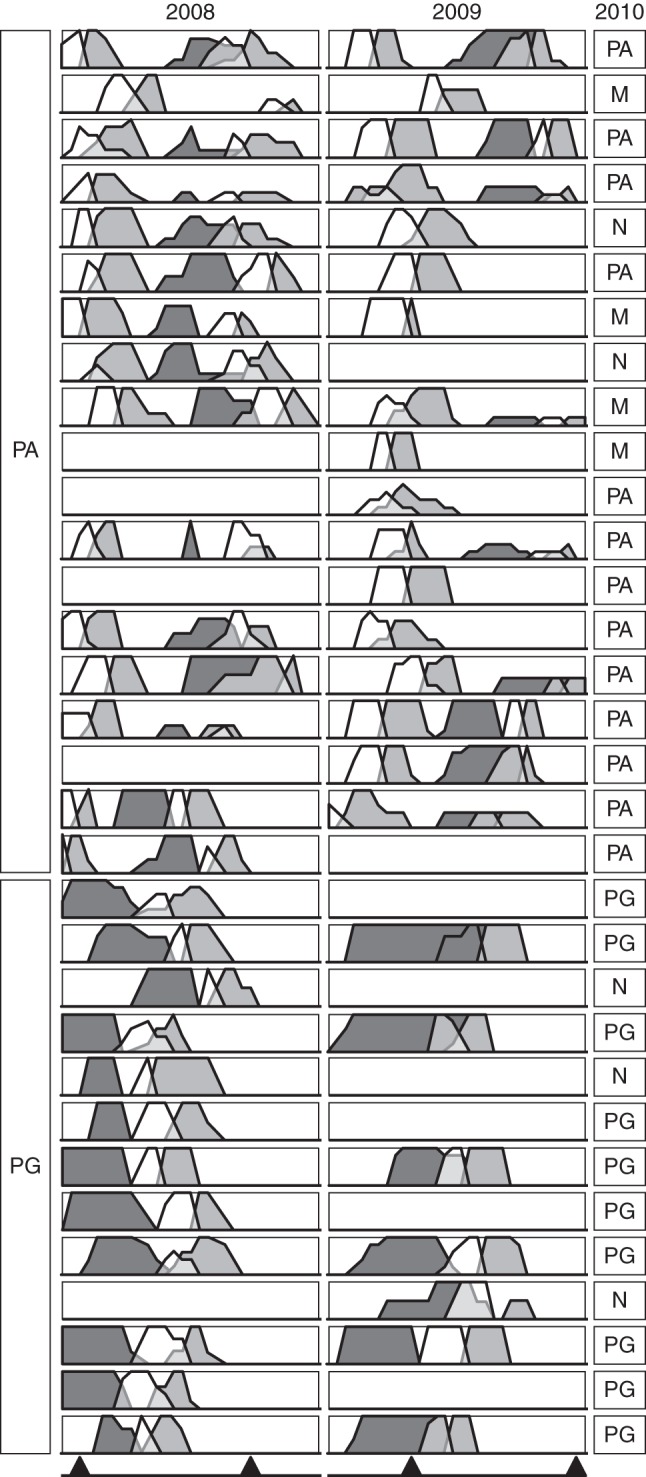
Phenology of 19 protandrous (PA) and 13 protogynous (PG) *Platycarya strobilacea* trees. Each row represents a tree, and each shaded line indicates the proportion of inflorescences in two consecutive male stages, M1 (white) and M2 (light grey), and female stage (F1, dark grey) over time in 2008 (left) and 2009 (right). In each male spike or male portion of an androgynous spike, showy and scent-emitting stage M1 is followed by less attractive stage M2 (for detailed definition of the stages, see Table 1). Blank cells represent non-flowering trees. Black triangles along the bottom indicate 5 June and 25 June. Along the right margin are indicated the flowering/sexual status of the trees in 2010 by PA (protandrous), PG (protogynous), M (male) and N (non-flowering).

The temporal changes in sex expression of 19 PA and 13 PG trees were typical of heterodichogamous populations (Fig. 4). All the trees were dichogamous at an individual level; inflorescences of each tree are synchronous as for male or female stages and each tree functioned mostly as either male or female at any given time. Within each tree, the M1 male phase tended to overlap with the preceding F1 female stage, while M2 was temporally distinct, except for several PG trees in 2009. Within each morph, the male and female stages were somewhat synchronous among trees. The male stages of one morph tended to coincide with the female stages of the other. When PA trees were functionally male, their male stages were synchronous with the first male stages of monoecious PA trees.

Among the 32 monitored trees, 27 flowered in 2008, 24 in 2009 and 27 in 2010 (Fig. 4, Table 2). Only 13 (seven PA, six PG) trees set monoecious inflorescences every year, and the others either flowered as functional males or not at all in 1 or 2 years. Of the 14 PA trees that were monoecious in 2008, eight were also monoecious in 2009, four were functionally male and two did not flower. Of the 12 PG trees that were monoecious in 2008, six were also monoecious in 2009, and six did not flower in 2009. Two relatively small (<3 m in height) trees were either non-flowering or functionally male from 2008 to 2010. No trees changed from PA to PG or vice versa in 3 years.

**Table 2.** Sexual status of 32 monitored *Platycarya* trees from 2008 to 2010; the trees were individually marked and followed across three years (see also Fig. 4)

Morph	Sex	2008	2009	2010
Protandrous (*n* = 19)	Monoecious	14	9	13
	Male	1	8	4
	Non-flowering	4	2	2
Protogynous (*n* = 13)	Monoecious	12	7	10
	Male	0	0	0
	Non-flowering	1	6	3

**Fig. 5.**
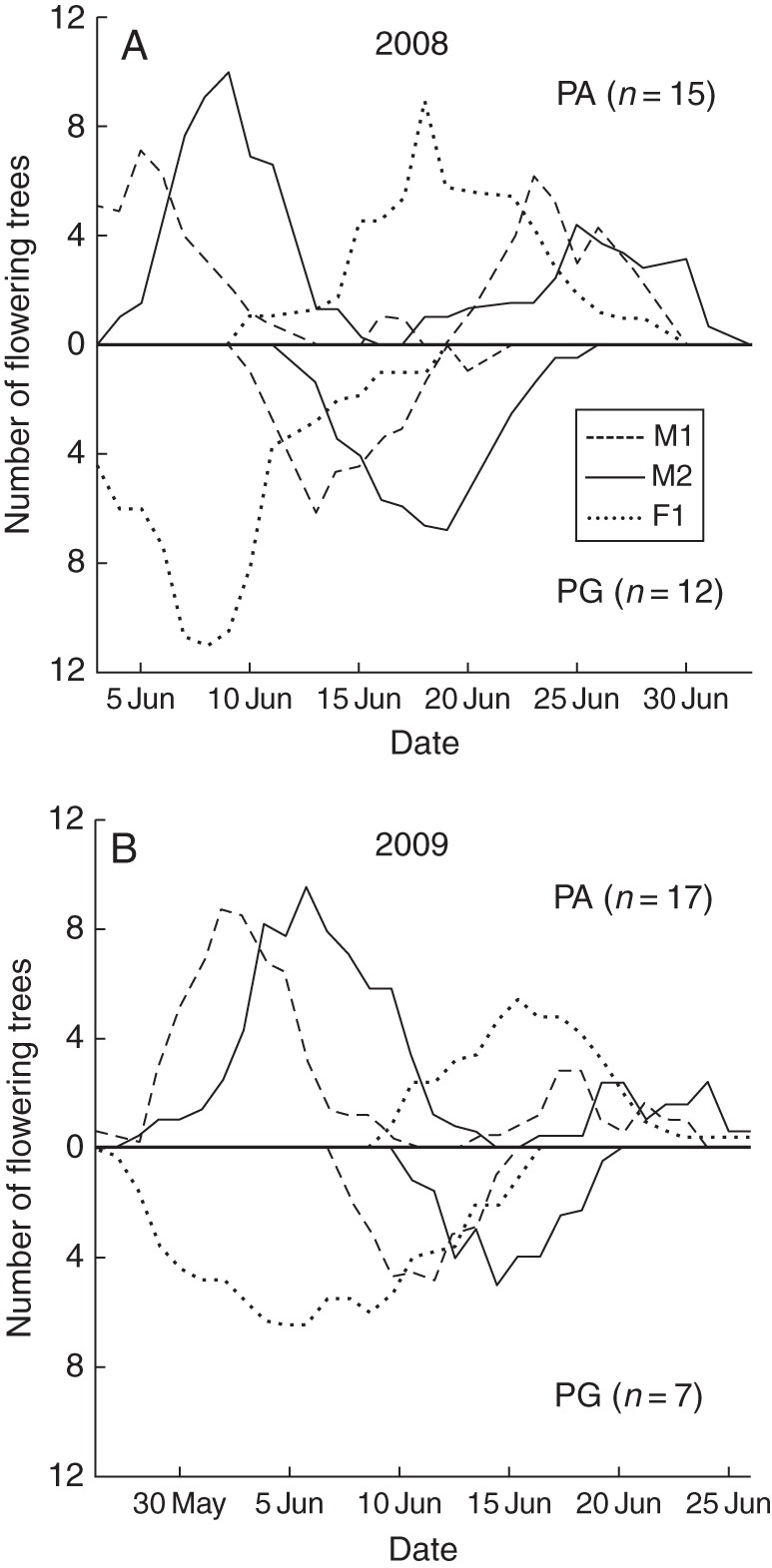
Temporal changes of the estimated number of *Platycarya strobilacea* trees in male (M1 and M2) and female (F1) stages in (A) 2008 and (B) 2009. Curves of protandrous (PA) trees, including functional males, are shown above and those of protogynous (PG) trees are shown below the central horizontal axis. In each male spike or male portion of androgynous spike, the showy and scent-emitting stage M1 is followed by the less attractive stage M2. For detailed definition of the stages, see Table 1.

The average length of the male phase of a spike was 6·4–7·5 d, and that of the female phase was 5·0–12·2 d (Table 3). Female stages tended to be longer in PG than in PA morphs and longer in 2009 than in 2008. Reciprocal transitions between PA and PG morphs were also evident at the population level (Fig. 5). The M2 stages were more synchronous with the F1 stages than were the M1 stages.

**Table 3.** *Duration of male (M1, M2) and female (F1) stages of protandrous (PA) and protogynous (PG) inflorescences of*Platycarya strobilacea

		PA					PG		
		Main axis		Lateral branches			Main axis		
		M1	M2	F1	M1	M2	F1	M1	M2
2008	Duration (d)	2·6 ± 1·0	3·8 ± 1·4	5·0 ± 2·8	2·7 ± 1·3	2·8 ± 1·4	7·2 ± 2·0	2·8 ± 1·5	3·9 ± 1·5
	*n*	56	59	50	47	48	38	37	37
2009	Duration (d)	3·1 ± 1·1	4·2 ± 1·6	7·7 ± 1·9	2·3 ± 0·9	2·6 ± 1·2	12·2 ± 3·8	3·4 ± 1·0	4·1 ± 1·2
	*n*	69	69	29	29	29	26	26	26

Values for duration are mean ± s.d. of *n* samples.

**Fig. 6.**
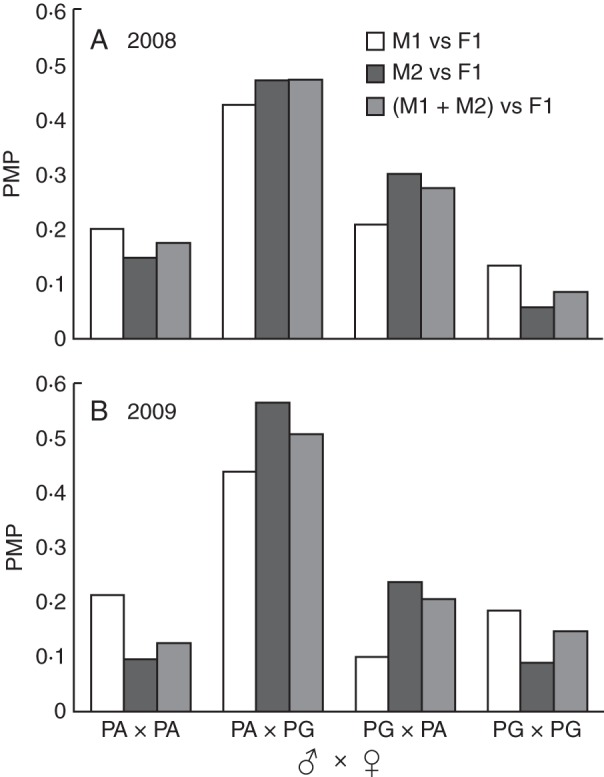
Potential mating probabilities (PMPs) of *Platycarya strobilacea* calculated from the phenological data in (A) 2008 and (B) 2009. The key indicates data for PMP when M1 stages were counted, when M2 stages were counted, and when both M1 and M2 stages were counted.

As expected from the within-morph synchronicity and between-morph reciprocity, the PMPs of between-morph pollination were higher than those of within-morph pollination (Fig. 6). The difference was larger when M2 was counted (0·76 versus 0·20 in 2008, 0·80 versus 0·20 in 2009) than when both M1 and M2 were counted (0·74 versus 0·26 in 2008, 0·71 versus 0·27 in 2009). When M1 was counted, the difference was smallest (0·63 versus 0·33 in 2008, 0·54 versus 0·40 in 2009).

**Fig. 7.**
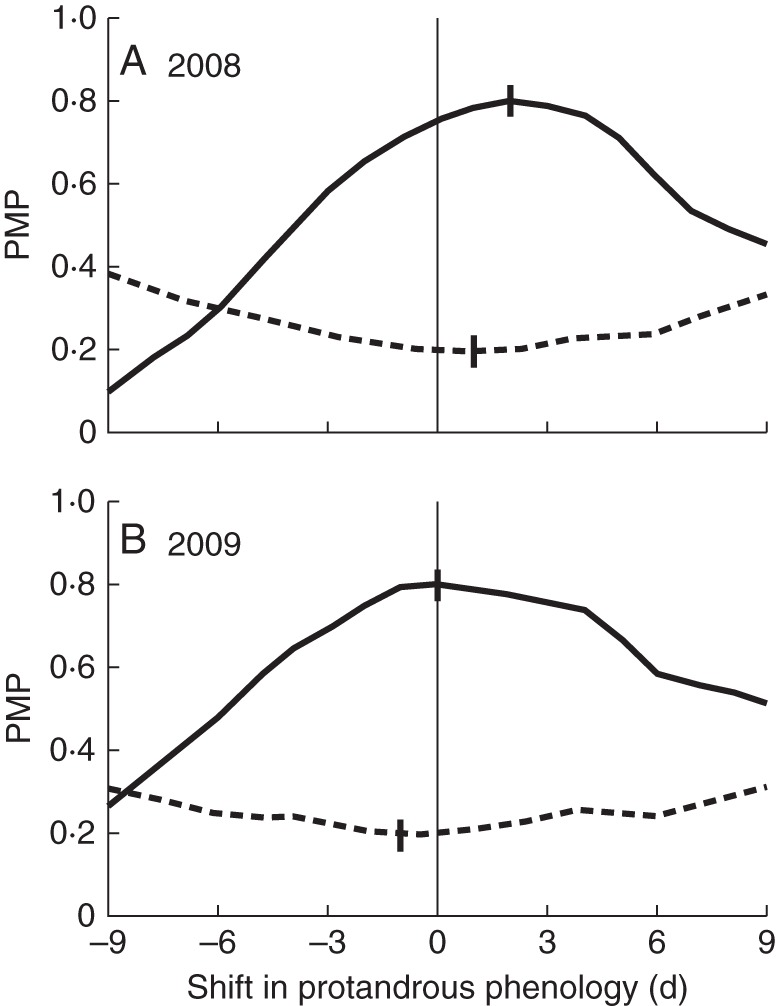
Potential mating probabilities (PMPs) of *Platycarya strobilacea* between-morph (solid line) and within-morph (dotted line) pollination calculated from the observed and simulated phenologies in (A) 2008 and (B) 2009. In the calculations, M2 stages were counted as male. The maximum or minimum is marked with a vertical bar.

Treating M2 as the male stage of pollen export, simulated PMPs were calculated in which the phenology of the PA morph was shifted 1–9 d earlier or 1–9 d later than the observed phenology. Comparing observed and simulated PMPs showed that the observed relative timings of PA and PG phenology were within 2 d of the optimum that maximized the opportunity for between-morph pollination and minimized that for within-morph pollination in both 2008 and 2009 (Fig. 7).

### Flower visitors/inhabitants

Aside from thrips, only 16 (11 species) and two (two species) insects were observed to land on flowering spikes during 50 daytime and 5 night-time hours of observation, respectively. No insect other than thrips showed recognizable pollinating behaviour, e.g. foraging for pollen or touching stigmas.

**Fig. 8.**
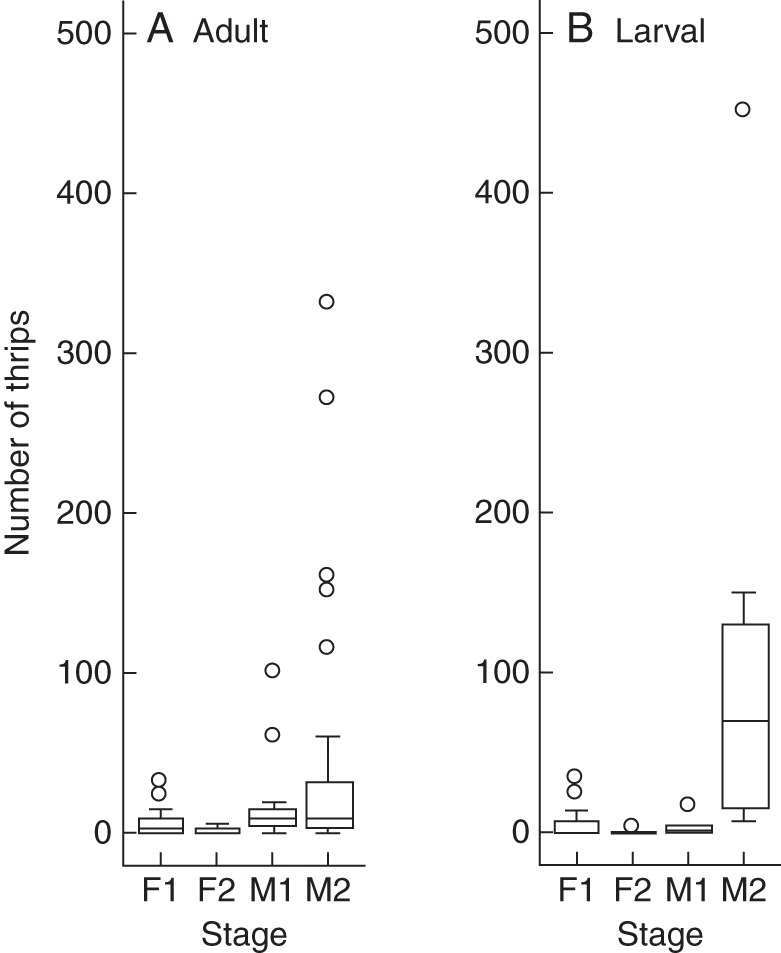
Boxplot diagrams showing the number of (A) adult and (B) larval thrips per five spikes in male and female stages of *Platycarya strobilacea*. The bottom and top of each box are the lower and upper quartiles, respectively, and the horizontal bar is the median. The upper and lower ends of the whiskers represent the maximum and minimum, respectively, except for outlier values indicated by open circles.

**Fig. 9.**
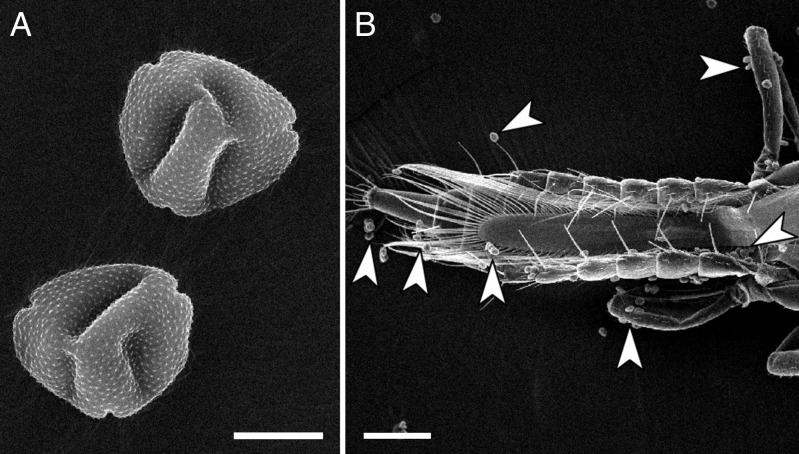
Scanning electron micrographs of pollen grains and thrips. (A) Pollen grains of *Platycarya strobilacea*. (B) Abdomen of a thrips collected from a male spike of *P. strobilacea*. Pollen grains are indicated by arrowheads. Scale bars: A, 10 µm; B, 100 µm.

Both adult and larval thrips were frequently found in male and female spikes or portions (Table 4; Fig. 8). They included *Ernothrips lobatus* Bhatti, *Thrips coloratus* Schmutz, *Thrips hawaiiensis* (Morgan) and *Haplothrips nipponicus* Okajima (M. Masumoto, Yokohama Plant Protection Station, The Ministry of Agriculture, Forestry and Fisheries of Japan, pers. comm.). Pollen grains (Fig. 9A) were observed on various body parts of thrips collected from male spikes (Fig. 9B).

**Table 4.** Numbers (mean ± s.d.) of adult and larval thrips per five spikes of *Platycarya strobilacea* at different plant stages

	M1	M2	F1	F2
No. of adults	17·6 ± 28·0	40·1 ± 73·6	4·7 ± 7·0	1·3 ± 2·0
*n*	13/14	37/38	22/36	3/7
No. of larvae	3·4 ± 5·8	92·3 ± 115·3	5·6 ± 10·1	0·4 ± 1·1
*n*	4/7	14/14	8/16	1/7

*n*, number of five-spike samples with thrips/total number of samples examined.

Thrips counts varied both among and within functional sex and stage (Table 4; Fig. 8). Thrips tended to be found more frequently and in larger numbers in male spikes than in female portions. More thrips were collected from M2 than from M1 spikes on average (3·5 adult and 0·7 larval thrips per spike in M1; 8·0 adult and 18·5 larval thrips per spike in M2). For adult thrips, the deviance was not significant (*P* = 0·1755) between the GLM considering the effect of stages (d.f. = 50, deviance = 62·794) and the null GLM (d.f. = 51, deviance = 65·658). For larval thrips, the deviance was significant (*P* = 0·0008) between the GLM considering the effect of stages (d.f. = 19, deviance = 24·655) and the null GLM (d.f. = 20, deviance = 45·346).

**Fig. 10.**
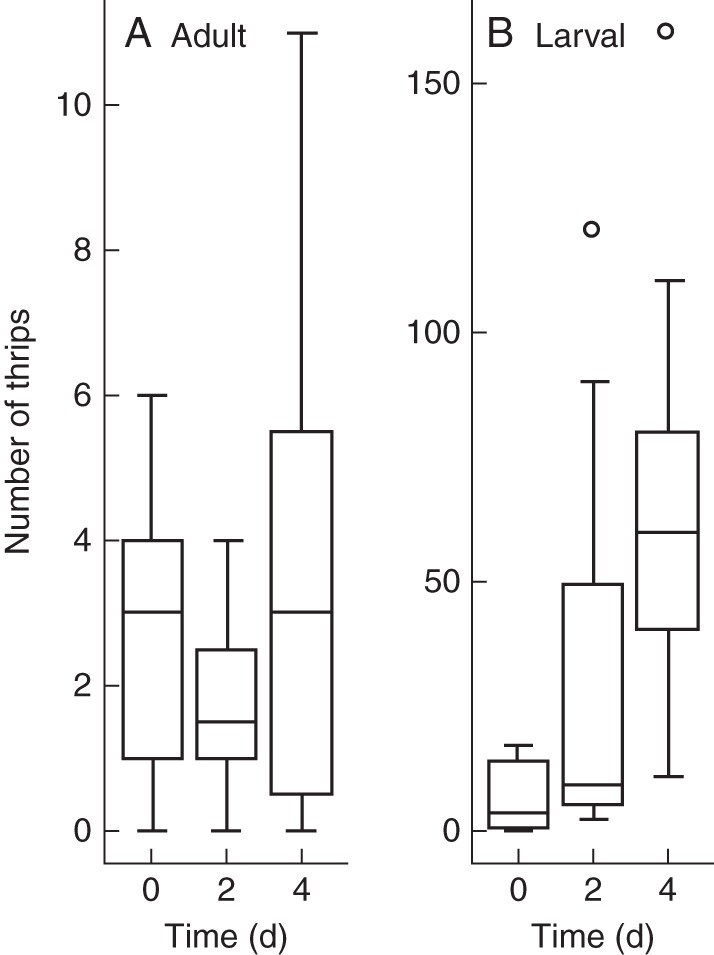
Boxplot diagrams showing the number of (A) adult and (B) larval thrips in a male spike of *Platycarya strobilacea* at M1 stage after 0, 2 and 4 d of storage. The bottom and top of each box are the lower and upper quartiles, respectively, and the horizontal bar is the median. The upper and lower ends of the whiskers represent the maximum and minimum, respectively, except for outlier values indicated by open circles.

After M1 spikes were stored in plastic bags for 0, 2 and 4 d, the numbers of larval and adult thrips, both in and out of spikes, were counted. Larval thrips increased rapidly in number (Fig. 10B), while adults did not (Fig. 10A). For adult thrips, the deviance was not significant (*P* = 0·5691) between the GLM considering the effect of storage period (d.f. = 34, deviance = 41·024) and the null GLM (d.f. = 35, deviance = 41·349). For larval thrips, the deviance was significant (*P* = 0·0001) between the GLM considering the effect of storage period (d.f. = 34, deviance = 42·041) and the null GLM (d.f. = 35, deviance = 64·051).

More thrips were collected from F1 than from F2 stages on average (0·9 adult and 1·1 larval thrips per spike in F1; 0·3 adult and 0·1 larval thrips per spike in F2). For adult thrips, the deviance was not significant (*P* = 0·0817) between the GLM considering the effect of stages (d.f. = 41, deviance = 43·620) and the null GLM (d.f. = 42, deviance = 46·166). For larval thrips, the deviance was significant (*P* = 0·0338) between the GLM considering the effect of stages (d.f. = 21, deviance = 17·779) and the null GLM (d.f. = 22, deviance = 21·493).

## DISCUSSION

### Heterodichogamy associated with inflorescence dimorphism

Distinguishing heterodichogamy from other polymorphic and/or labile sexual systems requires long-term monitoring of individuals (Renner *et al.*, 2007). Temporal changes in male and female maturity of 32 individually marked trees in 2008, 2009 and 2010 (Fig. 4, Table 2) indicated that the observed population of *Platycarya strobilacea* showed heterodichogamous phenology with PG (protogynous) and PA (protandrous–duodichogamous) morphs. No tree changed from one morph to the other during 3 years, suggesting that the sex morphs were genetically determined.

In *P. strobilacea*, functionally male trees are very likely to be an environmentally induced phenotype of PA rather than a separate morph. Functionally male inflorescences, which can be interpreted as PA inflorescences that lack lateral branches (Fig. 1), were often scattered among PA ones on the same tree. Male trees functioned as PA in some years and/or their male phase was synchronous with that of PA (Fig. 4). In contrast, PG trees scarcely bore functionally male inflorescences, and no tree functioned as PG in one year and as male in another (Fig. 4).

A prominent feature of the heterodichogamy of *Platycarya* is that it is associated with a distinct inflorescence dimorphism (Fig. 1). Both PG and PA morphs possess a monoecious module composed of a central androgynous spike and surrounding male spikes. A PG inflorescence is itself the module (Fig. 2A, B). In a PA inflorescence, the modules are lateral branches that flower after the terminal male spikes (Fig. 3A–C).

In previous morphological or floristic studies, the authors mainly described the characteristics of the PG morph. Some did not mention traits of the PA morph at all (Atkinson and Upson, 2006; Ohba, 2006), while others considered the PA inflorescence to be a male inflorescence resulting from the abortion of female flower clusters (Lu *et al.*, 1999; Li *et al.*, 2005) or a compound structure of several inflorescences (Manning, 1938).

A strikingly similar type of heterodichogamy has been reported for *Triadica sebifera* (L.) Small (= *Sapium sebiferum* L.; Euphorbiaceae) by Okamoto (1987). *Triadica* differs from *Platycarya* only in that both apical and lateral branches lack male spikes surrounding the central one. In both genera, the inflorescence dimorphism remains obvious during fruiting; the PG infructescence terminates the main axis (Fig. 2C), and the PA ones terminate the lateral branches (Fig. 3D). In other heterodichogamous taxa, including *Juglans* and *Carya*, no architectural differences between morphs have been reported, except for some *Acer* species in which the position of female flowers or fruits is somewhat different between PA and PG (Binggeli, 1990; Tal, 2009).

### Thrips pollination

Thrips-pollinated flowers or inflorescences have been reported from several angiosperm families and one cycad genus (Terry, 2001; Endress, 1994, 2010). Although our data cannot completely exclude the possibility of ambophily (Culley *et al.*, 2002), a combination of both thrips and wind pollination in this case, thrips pollination (thripophily) of *Platycarya* is suggested by (1) the frequent presence of thrips in spikes (Table 4; Fig. 8) and the scarcity of other visitors; (2) pollen grains attached to thrips' bodies (Fig. 9); (3) the synchronicity of thrips number in male spikes with the maturation of female flowers; and (4) morphological characters found in previously reported thrips-pollinated plants.

Male spikes at the M1 stage were bright yellow and emitted strong scents; both traits are frequently reported in thrips-pollinated flowers (Endress, 1994, 2010; Terry, 2001). Those at the M2 stage were less attractive, i.e. brownish with little or no scent. However, they contained more thrips (Table 4; Fig. 8) and synchronized better with the receptive F1 stage of female flowers of the reciprocal morph (Figs 4–6). Therefore, the M2 stage is probably the main period of pollen export, and there is probably a temporal decoupling of pollinator attraction and pollen export in male flowers of *Platycarya*.

Thrips-pollinated flowers tend to have narrow and relatively sheltered spaces where thrips propagate while eating pollen grains (Endress, 1994, 2010; Terry, 2001; Sakai, 2001, 2002). In male spikes of *Platycarya*, medium-sized and basally widened bracts are fused with the receptacle of the subtended male flower (Li *et al.*, 2005). The upper parts of the bracts are reflexed at anthesis but the basal parts remain upright. The stamen filaments are short and stout. These structures form narrow, hidden spaces around the axis near the anthers. The propagation of thrips in male spikes is suggested by the increase of the number of thrips with the stages (Table 4; Fig 8) and that in the stored male spikes (Fig. 10). The temporal decoupling of pollinator attraction and pollen export in male flowers might be explained if the thrips are attracted to the inflorescences in the M1 stage, and multiply sufficiently rapidly that offspring then carry the pollen from M2-stage inflorescences to female flowers.

Pollen grains of *Platycarya* are small enough to be carried on the body parts of thrips (Fig. 9). They are far smaller than those of other juglandoid genera (Endress, 1986; Manchester, 1989; Wang *et al.*, 1995). According to Wang *et al.* (1995), the pollen grains of *Platycarya* are approx. 20 µm in diameter along the long axis, while those of the other juglandoids range from 30 to 72 µm.

*Platycarya* is the second known species, in addition to *Acer pseudoplatanus* (Tal, 2009), with both heterodichogamy and thrips pollination. Thrips pollination of *Platycarya* probably derived from wind pollination because wind pollination is pervasive in the rest of Juglandaceae as well as closely related families (see Introduction). There are two suspected cases of the transition from wind pollination to thrips pollination. In *Thymelaea hirsuta*, thrips has been suggested to be an important pollinator in the northern edge of its distribution range, while wind pollination has been reported for non-marginal populations (Cornara *et al.*, 2005). In Moraceae, insect pollination has been suggested to be derived from ancestral wind pollination in the common ancestor of thrips-pollinated Castilleae and fig wasp-pollinated *Ficus* (Datwyler and Weiblen, 2004).

### Temporal sex expression and duodichogamous PA

While *Juglans* and *Carya* are self-compatible (Gleeson, 1982; Thompson and Romberg, 1985), we were not successful in testing whether *Platycarya* is self-compatible or not. Because M2 stage (indicated by light grey areas in Fig. 4) and F1 stage (dark grey areas) scarcely overlap each other within each tree, the opportunity of geitonogamous selfing seems very small. Theoretical models have predicted that heterodichogamy has an advantage over monomorphic synchronous dichogamy in that it avoids not only self-pollination but also biased temporal sex ratios during the flowering period (Wells and Lloyd, 1991; Sargent *et al.*, 2006). It requires a morph ratio close to 50 : 50 and exact reciprocity of temporal sex expression between PA and PG individuals at the population level. In fact, previous field studies have shown this condition in *Grayia* (Pendleton *et al.*, 1988), *Juglans* (Kimura *et al.*, 2003; Bai *et al.*, 2006) and *Kingdonia* (Wang *et al.*, 2012). In the observed population of *Platycarya*, 25 PA and 22 PG trees were observed (of these, temporal sex expression was monitored for 19 PA and 13 PG trees). The temporal change in tree numbers at the F1 stage of one morph was well synchronized with that of the M2 stage of the other, except for the later M2 peak of PA (Fig. 5). The PMPs between PA and PG were much higher than those within a morph (Fig. 6), and relative timings of PA and PG phenology approximately maximized the opportunity for between-morph pollination and minimized that for within-morph pollination (Fig. 7). Although we do not have genetic data to verify it, temporal patterns of sex expression strongly suggest that the heterodichogamous flowering system of *Platycarya* effectively enhanced mating opportunities between morphs and promoted outbreeding.

Protandrous trees of *Platycarya* are duodichogamous (flowering in male–female–male sequence; Lloyd and Webb, 1986; Luo *et al.*, 2007). In each tree, a large mass of male flowers blooms first, followed by the female flowers and then by a smaller mass of male flowers. A very similar condition has been reported in some *Acer* species (de Jong, 1976, 1994; for a review, see Renner *et al.*, 2007) and in *Triadica* (Okamoto, 1987). It is notable that *Acer*, *Triadica* and *Platycarya* are monoecious and insect-pollinated while six monoecious and wind-pollinated genera have simple protandrous PA morphs.

### Distribution of heterodichogamy in Juglandoideae

Recent phylogenetic studies have placed *Platycarya* in subfamily Juglandoideae with strong bootstrap support (Manos and Stone, 2001; Li *et al.*, 2004; Manos *et al.*, 2007; Sauquet *et al.*, 2012). The subfamily consisted of three well-supported clades, (1) *Platycarya*, (2) *Carya*–*Annamocarya* and (3) *Juglans*–*Pterocarya*–*Cyclocarya* (Manos *et al.*, 2007), and heterodichogamy has been reported from each of them (*Juglans*, *Carya* and *Platycarya*), suggesting it may be the ancestral condition of the subfamily.

Although inflorescence architecture is variable in Juglandaceae and has received much attention in evolutionary and systematic studies (Manning, 1938; Stone 1993; Manos and Stone, 2001), the inflorescence dimorphism associated with heterodichogamy of *Platycarya* has been long overlooked. Thus, further studies that intentionally focus on temporal flowering patterns in more taxa of Juglandaceae and related families, particularly Engelhardioideae, in which androgynous spikes are also found, will contribute to our understanding of the evolution of heterodichogamy as well as inflorescence structure in Juglandaceae.
